# Systematic review and meta-analysis determining the benefits of in vivo genetic therapy in spinal muscular atrophy rodent models

**DOI:** 10.1038/s41434-021-00292-4

**Published:** 2021-10-06

**Authors:** Ellie M. Chilcott, Evalyne W. Muiruri, Theodore C. Hirst, Rafael J. Yáñez-Muñoz

**Affiliations:** 1 AGCTlab.org, Centre of Gene and Cell Therapy, Centre for Biomedical Sciences, Department of Biological Sciences, School of Life Sciences and Environment, Royal Holloway University of London, TW20 0EX London, UK; 2 Department of Neurosurgery, Royal Victoria Hospital, Belfast, BT12 6BA UK; 3 Present Address: Institute for Women’s Health, UCL, 86-96 Chenies Mews, London, WC1E 6HX UK

**Keywords:** Genetic transduction, Gene expression

## Abstract

Spinal muscular atrophy (SMA) is a severe childhood neuromuscular disease for which two genetic therapies, Nusinersen (Spinraza, an antisense oligonucleotide), and AVXS-101 (Zolgensma, an adeno-associated viral vector of serotype 9 AAV9), have recently been approved. We investigated the pre-clinical development of SMA genetic therapies in rodent models and whether this can predict clinical efficacy. We have performed a systematic review of relevant publications and extracted median survival and details of experimental design. A random effects meta-analysis was used to estimate and compare efficacy. We stratified by experimental design (type of genetic therapy, mouse model, route and time of administration) and sought any evidence of publication bias. 51 publications were identified containing 155 individual comparisons, comprising 2573 animals in total. Genetic therapies prolonged survival in SMA mouse models by 3.23-fold (95% CI 2.75–3.79) compared to controls. Study design characteristics accounted for significant heterogeneity between studies and greatly affected observed median survival ratios. Some evidence of publication bias was found. These data are consistent with the extended average lifespan of Spinraza- and Zolgensma-treated children in the clinic. Together, these results support that SMA has been particularly amenable to genetic therapy approaches and highlight SMA as a trailblazer for therapeutic development.

## Introduction

Spinal muscular atrophy (SMA) is a neuromuscular disease chiefly characterised by degenerating alpha motor neurons (MNs) caused by defects in the gene *Survival Motor Neuron 1* [1]. SMA is the second most common autosomal recessive disease after Cystic Fibrosis [2] and is also the most common genetic disease resulting in infantile death [3]. MN loss results in atrophy of skeletal muscles, paralysis and denervation of neuromuscular junctions [3]. SMA mostly affects children, with symptoms including muscle weakness, areflexia, difficulty swallowing and feeding, and in the most severe cases is fatal, with infantile death most commonly attributed to respiratory failure [4]. Although MNs are the cells primarily affected in this disease, systemic pathology exists. Muscular [5], vascular [6] and cardiac defects [7] have been reported.

95% of SMA patients show deletions of *SMN1*, with the remaining 5% carrying mutations in this gene. Homozygous deletions or mutations lead to no SMN protein production from *SMN1*, however this can be partially compensated for by the duplicated *SMN2* gene. Within *SMN2*, a C to T mutation 6-bp into exon 7 preferentially results in an alternatively spliced transcript lacking exon 7, known as *SMNΔ7*, which, when translated, leads to a truncated protein rapidly degraded. *SMN2* produces a small amount of full-length transcript and hence protein. The number of *SMN2* copies correlates inversely with the severity of SMA [8].

Two SMA treatments, Spinraza and Zolgensma, have been approved for marketing by the US Food and Drug Administration (FDA) and European Medicines Agency (EMA) within the last few years. Spinraza is an antisense oligonucleotide targeting *SMN2* splicing, aiming to promote inclusion of exon 7 within transcripts and hence the synthesis of full-length SMN protein. Spinraza binds to *SMN2* pre-mRNA at an intronic splicing sequence in intron 7, preventing negative splice factors from binding this site. This causes recognition of exon 7 by U1snRNP and inclusion in the mature *SMN2* mRNA transcript [9]. Zolgensma is a self-complementary AAV9 vector encoding *SMN1*. This therapy aims to replace the missing *SMN1* gene in SMA patients, thus restoring normal SMN protein function [10]. Both of these therapies were extensively tested in pre-clinical experiments before progressing to clinical trials. The approval of Spinraza was largely underpinned by data from ENDEAR and CHERISH clinical trials, whist only the START clinical trial using Zolgensma was completed prior to licensing. It is important to state that, technically, oligonucleotides are not classed as gene therapies by FDA or Advanced Therapy Medicinal Products by the EMA, while viral vectors like Zolgensma are. We therefore refer to both of them as “genetic” therapies in the current analysis. One more treatment for SMA, Risdiplam (Evrysdi) has been recently approved by both FDA and EMA. It is based on a small molecule able to alter the splicing of *SMN2* [11]; not being a genetic therapy, we will not discuss it further.

Here, we review all studies that have used a genetic therapy approach to treat SMA rodent models using meta-analytic techniques to provide quantitative data pertaining to treatment efficacy. This information is useful as it can provide insights into the most successful strategies in pre-clinical research, avoiding unnecessary and unethical repetition of animal experimentation [12], and identify gaps in knowledge that can be addressed in the future. Potential sources of bias and heterogeneity within pre-clinical studies were also explored. We discuss how effectively pre-clinical data can predict clinical trial outcome.

## Data source and analytical methods

### Study identification

The electronic databases PubMed and Web of Science were searched for relevant published studies between 1950 and 12th June 2020. Keyword strings “gene therapy AND spinal muscular atrophy” and “antisense oligonucleotide AND spinal muscular atrophy” were used. Despite literature often colloquially referring to the use of oligonucleotide-based approaches in gene therapy experiments, oligonucleotides are not officially classed as gene therapies by the FDA, or Advanced Therapy Medicinal Products by EMA. Therefore, the term genetic therapies has been used for the remainder of this analysis, with the exception of the search criteria. All languages were included in the search. No restriction concerning type of publication was used. Manual searching of the bibliographies of each of the electronically identified studies revealed references for additional studies which were then retrieved.

### Study selection criteria

Primary studies found from the electronic and manual searches were screened for eligibility based on the following inclusion criteria [1]: genetic therapy was administered in vivo [2]; a rodent model of SMA was used [3]; median survival data was reported in text, or was calculable form Kaplan–Meier plots; and [4] the number of animals in control and treated groups were reported. Here, in vivo genetic therapy was defined as the introduction of genetic material (DNA, RNA, oligonucleotides, viral vectors, bacterial vectors or genome editing technology) directly into an animal. All studies using pharmacological means to manipulate gene expression, for example histone deacetylase inhibitors or compounds such as Branaplam and Risdiplam, were excluded. No restrictions on the type of SMA rodent model were enforced.

### Data extraction of primary studies

Survival data and aspects of experimental design for each comparison were extracted from included publications. Experimental design characteristics included the type of genetic therapy agent used, rodent model, therapeutic target, delivery route and time of administration. Here, P1 was designated as the day of birth. Disparity was observed in the reporting of viral vector dose, with some studies using the total number of vector genomes (vg) administered per animal and others using vector genomes per kilogram (vg/kg). Here, all doses were converted to vg/kg using an approximate birth weight of 1 g per pup. Outcome data were recorded as median survival (the number of days at which 50% of animals were alive), and the number of animals in both control and experimental cohorts was recorded. If no median survival data were reported in the text, this was calculated from figures or sought through direct contact with authors. If more than 50% of animals survived at the end of the reported time period, the median survival value was recorded as the last time point of assessment. If studies presented multiple control groups, the following hierarchy was implemented and data were extracted from that of the highest relevance [1]: reporter gene (if viral vector) or scrambled ASO (if oligonucleotide) [2]; sham surgery or saline injection [3]; untreated. If data were presented from both heterozygous and homozygous SMA animal models, data were extracted from that of homozygous comparisons. If any data were not reported within the study, or if clarification was necessary, study authors were contacted; if no reply was received after two weeks, the relevant studies were excluded.

### Data analysis

Standard meta-analysis techniques could not be employed here given that no standard error or deviation is associated with median survival data. Therefore, the meta-analysis workflow used here was adapted using techniques presented in [12]. This approach has proven successful in other recent pre-clinical meta-analyses [13] and has shown to be comparable to standard (hazard or odds ratio) techniques [14].

Median survival ratios (MSR), equivalent to the survival of treated animals divided by survival of control animals, were calculated to summarise the median survival data that were extracted. This approach was used to maximise consistency with the hazard ratio method commonly used in meta-analyses [14, 15]. Log-transformed MSRs were entered into a random effects model adapted from DerSimonian and Laird [16, 17] with the number of animals used as a measure of precision to weight each study. The number of animals was calculated as the sum of treated and true control (number of control animals divided by the number of treatment groups per control group) animals. To achieve an estimate of variance from data that does not contain an inherent error or deviation value, a fixed effect size with associated measure of heterogeneity, denoted by tau, was first calculated. This was then substituted into the random effects model. Finally, the overall MSR (a measure of whether treatment provided a therapeutic benefit or not) was calculated with associated 95% confidence intervals and a final random effects standard error. An MSR of 1 represents a neutral treatment effect, <1 suggested genetic therapy was detrimental to survival, >1 suggests genetic therapy provided a survival advantage.

A stratified meta-analysis was undertaken so that the effect of different experimental intervention conditions could be analysed. The effect of heterogeneity across strata was identified using the *χ*^2^ statistic to determine a threshold level of significance to compare all individual stratifications to. Seven strata were used in this review; type of genetic therapy, dosage of viral vector, overall therapeutic target and *SMN1*- versus *SMN2*-based approaches, mouse model and finally route and time of administration. The *χ*^2^ statistic with degrees of freedom equal to the number of sub-strata minus 1 was adjusted to account for stratifications using the Bonferroni correction. The threshold level of significance calculated was equal to *P* = 0.0073. Stratifications that produced a Bonferroni adjusted *P* value less than *P* = 0.0073 suggested that heterogeneity between sub-strata, and thus the MSR, was significantly different from one another.

Publication bias was assessed using funnel plots, Egger regression [18] and Trim and Fill analysis [19] using the number of animals as a measure of precision. The number of animals, instead of inverse variance, as previously described [12, 13] avoids potential correlation between standard error and effect sizes that can cause the appearance of funnel plot asymmetry [20].

#### Software

Searches were uploaded to the Collaborative Approach to Meta-Analysis and Review of Animal Data from Experimental Studies tool to screen studies for inclusion or exclusion. Data extraction and statistical calculations were performed in Microsoft Excel and Stata. Graphical results were created using the ggplot2 package within R and Microsoft Excel.

## Results

### Publication identification

**Fig. 1:**
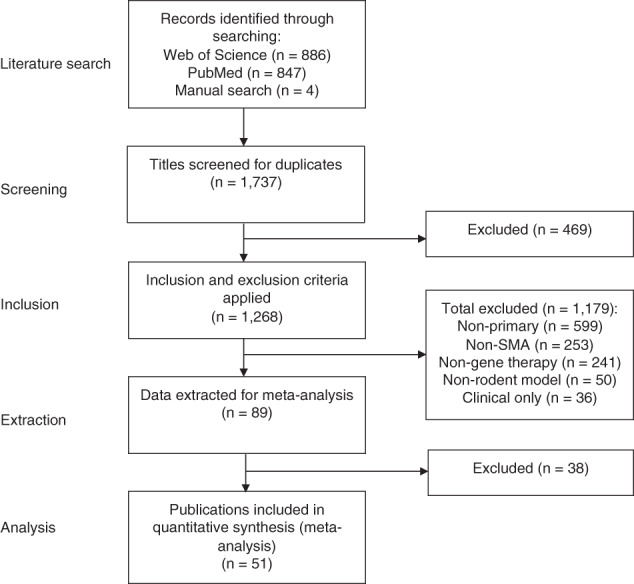
Flow chart illustrating steps in study identification and assessment of eligibility for inclusion in the meta-analysis. *n* number of studies.

**Fig. 2:**
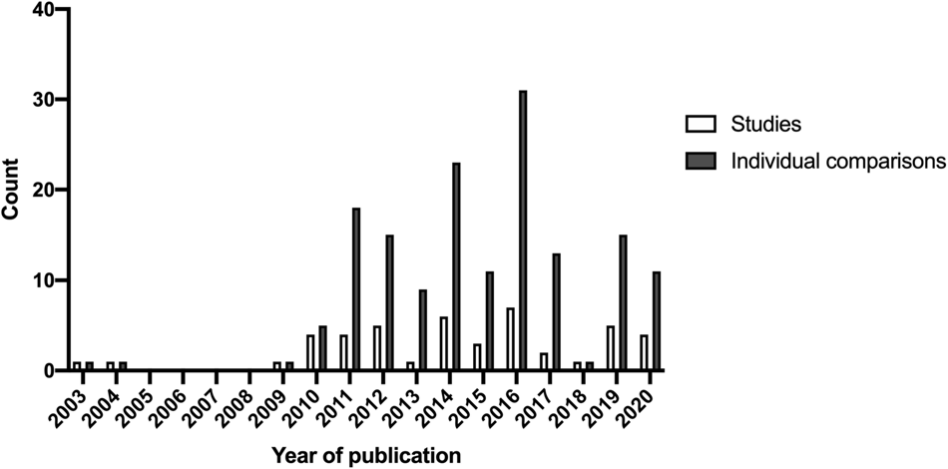
Distribution of studies and the individual comparisons they contain. 51 eligible studies were included in this meta-analysis. Some publications contained multiple comparisons within the main study; together 155 individual comparisons were assessed here.

Electronic and manual searching retrieved 1737 publications, 469 of which were duplicates found from more than one database search. 1268 publications were screened to determine if they met inclusion criteria, 1179 of these were excluded. Reasons for exclusion included: reviews or non-primary literature, non-SMA, non-genetic therapy intervention, non-rodent model, clinical only data. Of the 89 publications that were deemed relevant, data extraction was completed successfully for 51 [21–71]. The remaining 38 were excluded due to missing, incalculable or irrelevant data. From the included publications, 155 individual comparisons were used in statistical analysis, corresponding to 2573 animals in total. This information is summarised in Fig. 1, with characteristics of included publications in Table 1. Figure 2 shows the distribution of publications and individual comparisons across the years, highlighting a large increase from 2009 onwards.

**Table 1:** Overview of pre-clinical gene therapy applications in SMA mouse models.

Study	Genetic therapy	Target and/or transgene	SMA model	Administration		Dosage	Median survival (days)	
				Time	Route		Treated	Control
Oligonucleotide-based approaches:								
Baughan et al. [21]	RNA	*SMN2*	Burghes’ severe	P2 + P4	ICV	6 μg	7	5
Coady and Lorson [22]	ASO	*SMN2*	Burghes’ severe	P1	ICV	Not reported	7	4
Shababi et al. [23]	RNA	*SMN2*	Burghes’ severe	P2 + P3 + P4	ICV	10 μg	6	4
		*IGF1*					7	
		*SMN2* + *IGF1*					8	
Hua et al. [24]	ASO	*SMN2*	Taiwanese	P1	ICV	20 μg	16	10
				P1 + P3	SC	50 μg/g	108	9
				P1 + P3 + P5 + P7	SC	50 μg/g	137	9
				P1 + P3	ICV + SC	20 μg + 50 μg/g	173	10
				P1 + P3	SC	40 μg/g	84	10
				P1 + P3	SC	80 μg/g	170	10
				P1 + P3	SC	160 μg/g	248	10
				P5 + P7	SC	100 μg/g	16	11
				P1 + P3	IP	80 μg/g	118	11
Passini et al. [25]	ASO	*SMN2*	SMNΔ7	P1	ICV	8 μg	23	16
						4 μg	25	
						2 μg	23	
						1 μg	20	
						0.5 μg	17	
Osman et al. [26]	ASO	*SMN2*	SMNΔ7	P1	ICV	6 μg	16.5	12
	RNA 1						19	
	RNA 2						20	
Porensky et al. [27]	MO	*SMN2*	SMNΔ7	P1	ICV	27 μg	83	15
					ICV	54 μg	104	
					ICV	81 μg	112	
					IV	50 μg/g	35	
					IV + ICV	54 μg	93	
				P4	ICV	54 μg	41	
				P4	IV	81 μg	21	
Zhou et al. [28]	PMO 18	*SMN2*	Taiwanese	P1	ICV	20 μg/g	12	9.5
						40 μg/g	32	
	PMO 25					20 μg/g	43	
						40 μg/g	85.5	
					IV		230	
				P1 + P3	IV + SC/IP	40 μg/g	93.5	
	VPMO 25				IV + IP	7 μg/g	16	
Keil et al. [29]	ASO	*SMN2*	Taiwanese	P1 + P5 + P10	IP	20 μg/g	8	10
						80 μg/g	13	10
			Hemi-hybrid			80 μg/g	50	16
Nizzardo et al. [30]	MO (modified)	*SMN2*	SMNΔ7	P1 + P3	ICV + SC	2 nM	42.5	17
	MO (unmodified)					10 nM	40	
						5 nM	46	
						2 nM	40	
Osman et al. [31]	ASO	*SMN2*	SMNΔ7	P2	IP	2 mM	14	10
					ICV		39	
					ICV + ICV		54	
			SMNRT		ICV + IP		54	
					ICV		175	17
Bogdanik et al. [32]	ASO	*SMN2*	II/III Burgheron	P10	IP	80 μg/g twice	169.5	125.5
				P25			100	
Hua et al. [33]	ASO	*SMN2*	Taiwanese	P1 + P3	SC	120 mg/kg	237	10
					ICV + SC	120 mg/kg + 30 μg decoy	212	10
Zhou et al. [34]	PMO 25	*SMN2*	Taiwanese	P1	ICV	40 μg/g	212	9.5
					SC		261	
					ICV	20 μg/g	43	
					SC		58	
					ICV	10 μg/g	22	
					SC		25	
Olivan et al. [35]	Plasmid	*SMN*	Type II	P1	IM	50 μg in two muscles	8	8
Hammond et al. [36]	PMO	*SMN2*	Taiwanese	P1	IV	10 μg/g	167	12
Hosseinibarkooie et al. [37]	ASO	*SMN2*	Taiwanese	P1 + P2	SC	30 μg	25	12.5
Lin et al. [38]	ASO	*SMN2*	Taiwanese	P1	SC	80 μg/g	19.7	7.7
Osman et al. [39]	ASO E1 MO	*SMN2*	SMNΔ7	P1	ICV	2 μl of 40 nM	47.8	12.3
	ASO E1^MOv1^						15.8	
	ASO E1^MOv2^						10.2	
	ASO E1^MOv3^						19	
	ASO E1^MOv4^						19.5	
	ASO E1^MOv5^						15.3	
	ASO E1^MOv6^						18.8	
	ASO E1^MOv7^						15.8	
	ASO E1^MOv8^						18.3	
	ASO E1^MOv9^						17.5	
	ASO E1^MOv10^						30.8	
	ASO E1^MOv11^						50.9	
	ASO E1^MOv12^						19.2	
Arnold et al. [40]	ASO	*SMN2*	SMNΔ7	P4	ICV	40 μg	60	16.5
				P6			22	
Riessland et al. [41]	ASO	*SMN2*	Taiwanese	P1	SC	30 μg	180	17
Shabanpoor et al. [42]	PMO (naked)	*SMN2*	Taiwanese	P1 + P2	IV	10 μg/g	29	14
	PMO (Br-ApoE)						78	
d’Ydewalle et al. [43]	ASO	*SMN2*	SMNΔ7	P1 + P3	SC	400 mg/kg	18	18
	SSO					50 mg/kg	25	
	ASO + SSO					400 mg/kg + 50 mg/kg	37	
Viral vector-based approaches:								
Lesbordes et al. [44]	Ad	*CT1*	NSE-Cre+ Smn^F7/F7^	P5–7	IM	10e8 pfu/mouse	44.4	33.7
Azzouz et al. [45]	EIAV SIN LV	*SMN1*	SMNΔ7	P2	IM	1.2e8 vg/mouse	18	13
Passini et al. [46]	ssAAV8	*SMN1*	SMNΔ7	P1	ICV + IS	5e10 vg/mouse	50	15
	scAAV8					1.7e10 vg/mouse	157	16
Valori et al. [47]	scAAV9	*Codon optimised SMN1*	SMNΔ7	P1	IV	10e11 vg/mouse	69.1	11.2
Foust et al. [48]	scAAV9	*SMN1*	SMNΔ7	P1	IV	5e11 vg/mouse	250	15.5
Dominguez et al. [49]	scAAV9	*SMN1*	SMNΔ7	P1	IV	4.5e10 vg/mouse	160	13.7
Glascock et al. [50]	scAAV9	*SMN1*	Burghes’ severe	P1	ICV	2e11 vg/mouse	17	7
					IV		10	
Glascock et al. [51]	scAAV9	*SMN1*	SMNΔ7	P2	IV	2e10 vg/mouse	34.9	11
				P2 + P3	ICV		126.7	
Shababi et al. [52]	scAAV9	*SMN1*	SMNΔ7	P2	IV	1e11 vg/mouse	23.5	12
Benkhelifa-Ziyyat et al. [53]	scAAV9	*Codon optimised SMN1*	SMNΔ7	P1 + P2	IM (2 limbs)	5e13 vg/kg	26	12
					IM (4 limbs)		163	
Tsai et al. [54]	AAV1	*IGF1*	Burghes’ severe	P1	IV	3.4e9 vg/mouse	12	9
Passini et al. [55]	scAAV9	*SMN1*	SMNΔ7	P1	ICV + IT	5e10 vg/mouse	153	17
						1e10 vg/mouse	70	
						1e9 vg/mouse	18	
Robbins et al. [56]	scAAV9	*SMN1*	SMNΔ7	P2	ICV	1e11 vg/mouse	204	14
				P3			75	
				P4			167	
				P5			37	
				P6			34	
				P7			28	
				P8			18	
Little et al. [57]	scAAV9	*PTEN*	SMNΔ7	P1	IV	10e10 vg/mouse	23.5	10
Powis et al. [58]	ssAAV9	*Uba1*	Taiwanese	P1	IV	2.4e11 vg/mouse	12	9
Odermatt et al. [59]	scAAV9	*SMN2 via U7-ESE-B*	SMNΔ7	P1 + P2	ICV	4.07e12 vg/kg	22	12
						1.75e13 vg/kg	25.5	
						3.21e13 vg/kg	33	
						4.34e13 vg/kg	34	
						2.27e14 vg/kg	195	
Armbruster et al. [60]	scAAV9	*Codon optimised SMN1*	SMNΔ7	P1	ICV	1.9e13 vg/kg	201	16
						3e13 vg/kg	346	
						7.5e13 vg/kg	154	
						1.9e13 vg/kg	283	
					ICV + IV	3e13 vg/kg	188	
						7.5e13 vg/kg	262	
Alrafiah et al. [61]	ssAAV9	*Plastin3*	SMNΔ7	P1	IT	5e10 vg/mouse	17.5	12.5
Villalon et al. [62]	scAAV9	*Stathmin1*	Smn2B/−	P2	ICV	1e11 vg/mouse	27.02	19.04
Donadon et al. [63]	AAV9	*SMN2 via ExSpeU1s*	Taiwanese	P1 + P3	IP	1.5e12 vg/mouse	219	10
				P1			150	
				P1 + P3		1.5e11 vg/mouse	13.56	
Rashnonejad et al. [64]	ssAAV9	*SMN1*	SMNΔ7	E14–15	ICV	4e10 vg/mouse	63	12
	scAAV9						105	
Simon et al. [65]	scAAV9	*Stasimon*	SMNΔ7	P1	ICV	1e11 vg/mouse	15.03	14.12
Osman et al. [66]	scAAV9	*SMN1*	SMNΔ7	P2	ICV	1e11 vg/mouse	70	10
		*D. rerio Smn*					70	
		*X. laevis Smn*					38	
		*D. melanogaster Smn*					13	
		*C. elegans Smn*					11	
		*S. pombe Smn*					9	
		*SMN_236*					15	13
			Smn2B/−				36	25
Ahlskog et al. [67]	scAAV8	*Klf15*	Taiwanese	P1	IV	2e10 vg/mouse	13.8	12.82
						1e10 vg/mouse	7.88	
			Smn2B/−			2e10 vg/mouse	21.73	20.7
Besse et al. [68]	AAV9	*Codon optimised SMN1 (hSYN)*	SMNΔ7	P1	ICV	4.5e10 vg/mouse	15.5	16
						1.2e11 vg/mouse	39.5	
		*Codon optimised SMN1 (hPGK)*				4.5e10 vg/mouse	221	
					IV		142	
Nizzardo et al. [69]	AAV9	*Syt13*	SMNΔ7	P1	IM	5e10 vg/mouse	18	12
Combinatorial approaches:								
Kaifer et al. [70]	scAAV9	*Plastin3*	SMNΔ7	P1	IV	1e11 vg/mouse	15	15
	ASO	*Plastin3*			ICV	1 nmol	17	15
	ASO + scAAV9	*SMN2* + *Plastin3*			ICV + IV	1 nmol + 1e11 vg/mouse	14	15
	ASO	*SMN2*			ICV	2 nmol	20	17
	ASO + scAAV9	*SMN2* + *Plastin3*	Smn2B/−		ICV + IV	2 nmol + 1e11 vg/mouse	43.5	17
	scAAV9	*Plastin3*			IV	1e11 vg/mouse	43.75	30
	scAAV9	*Plastin3*			IV	3e11 vg/mouse	45	30
Zhou et al. [71]	AAV	*Myostatin*	Taiwanese	P1	SC	2.5e10 vg/mouse	12	10
	PMO 25	*SMN2*				40 μg/g	261	
	PMO + AAV	*SMN2* + *Myostatin*				40 μg/g + 2.5e10 vg/mouse	166	

*ASO* antisense oligonucleotide, *MO* morpholino, *PMO* peptide morpholino, *SSO* splice switching oligonucleotide, *AAV* adeno-associated viral vector, *ss* single astranded, *sc* self-complementary, *EIAV* equine infectious anaemia virus, *SIN* self-inactivating, *LV* lentiviral vector, *IV* intravascular, *IT* intrathecal, *ICV* intracerebral ventricular, *IS* intraspinal, *IM* intramuscular, *IP* intraperitoneal, *SC* subcutaneous, *P1* post-natal day 1, *vg* vector genomes, *pfu* plaque forming unit.

*N* = 23 publications contained comparisons using an oligonucleotide-based approach, *n* = 26 used a viral vector and *n* = 2 used a combination of both. Genetic therapy agents were delivered between P1 and P25 into a range of models including Taiwanese (*n* = 14 studies) and SMNΔ7 (*n* = 28 studies) mice, via local (intrathecal, intramuscular and intracranial), systemic (intravascular, subcutaneous and intraperitoneal), or multiple routes of administration. The different characteristics of each study provided the basis of the stratified meta-analysis.

On pooling the 155 comparisons in meta-analysis, we found SMA animals treated with a genetic therapy to survive over 3 times as long as controls (MSR: 3.23, 95% CI 2.75–3.79; *χ*^2^ = 2671.65, df = 154; *P* < 0.0073).

### Stratification of data

#### Type of genetic therapy

Three categories of genetic therapy agents were compared; oligonucleotide-based approaches including antisense oligonucleotides (ASO), peptide morpholinos and naked DNA/RNA, viral vector-based approaches including AAV, adenoviral and lentiviral vectors and oligonucleotide plus viral vector combinatorial approaches. Oligonucleotide-based approaches led to the development of Spinraza, whilst viral vector-based approaches, specifically AAV, led to the development of Zolgensma. Therefore, this allows direct comparison of the efficacy of two drugs’ rationale, and how successfully these translated to human clinical trials.

**Fig. 3:**
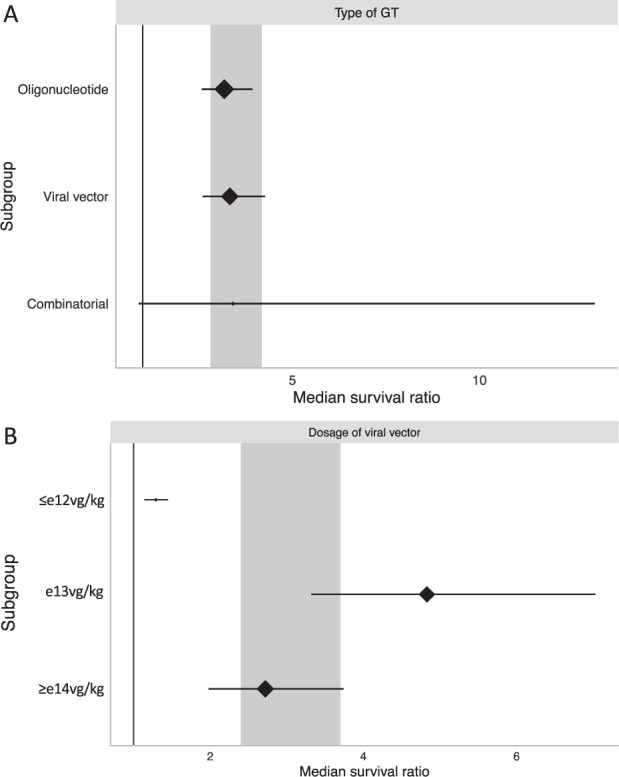
Stratification by type of gene therapy and dosage of viral vector. Both (**A**) type of gene therapy and (**B**) dosage of viral vector accounted for significant heterogeneity in median survival ratio (MSR *P* < 0.0073). **B** Sub-strata were defined as viral vector dosage of ≤e12, e13, and ≥e14 vg/kg. **A**, **B** Plots represent mean ± 95% confidence intervals with the size of diamonds representing the number of comparisons within each stratum. The vertical line at MSR = 1 represents a neutral treatment effect. Grey rectangles represent global 95% confidence intervals.

All three types of genetic therapy were associated with a significant increase in median survival (*χ*^2^ = 38.54, df = 2; *P* < 0.0073). Oligonucleotide approaches showed just over three-fold survival advantage (MSR: 3.18, 95% CI 2.58–3.93; *n* comparisons = 85; Fig. 3A) whilst viral vector approaches provided a similar increase (MSR: 3.33, 95% CI 2.60–4.27; *n* = 66; Fig. 3A). Efficacy was very similar, if slightly increased, when oligonucleotide and viral vectors were combined within a single treatment (MSR: 3.41, 95% CI 0.89–13.08; *n* = 3; Fig. 3A). However, only two publications [70, 71], containing three individual comparisons, used a combinatorial treatment so efficacy may be overestimated.

#### Viral vector dosage

Within the different types of genetic therapy, an attempt was made to further stratify by dosage of genetic therapy agent. This was possible for those studies that used viral vector-based approaches as raw data presented in total vector genomes per mouse, or vector genomes (vg)/kg could be delineated into discreet groups by conversion of all to vg/kg. However, dosage delineation for oligonucleotide-based approaches was not possible due to disparity in the presentation of dose. Some publications presented dose as weight-based measures (either absolute µg or µg/g) or in molar concentrations. Therefore, only sub-stratification by viral vector dosage is shown here.

Of the 69 comparisons that administered viral vectors, alone or in combination with an oligonucleotide, significant differences between efficacy were observed (*χ*^2^ = 1817.93, df = 2; *P* < 0.0073). A minority of comparisons used ≤e12 vg/kg leading to a small, but significant increase in survival (MSR: 1.29, 95% CI 1.14–1.45; *n* = 5; Fig. 3B). These comparisons either used ≤e12 vg/kg as a low dose comparison to others in e13 or ≥e14 vg/kg categories, or vector titre may have been limited due to the nature of virus used, as in the case of lentiviral vectors [45] and adenoviral vectors [44]. Approximately equal numbers of comparisons implemented either e13 or e14 vg/kg viral dosages. e13 vg/kg was associated with the largest survival advantage (MSR: 4.83, 95% CI 3.32–7.03; *n* = 30; Fig. 3B). Finally, the highest dose of viral vector (≥e14 vg/kg) produced a larger increase in survival than ≤e12 vg/kg, but not as high as e13 vg/kg (MSR: 2.72, 95% CI 1.98–3.74; *n* = 34; Fig. 3B).

#### Therapeutic target

**Fig. 4:**
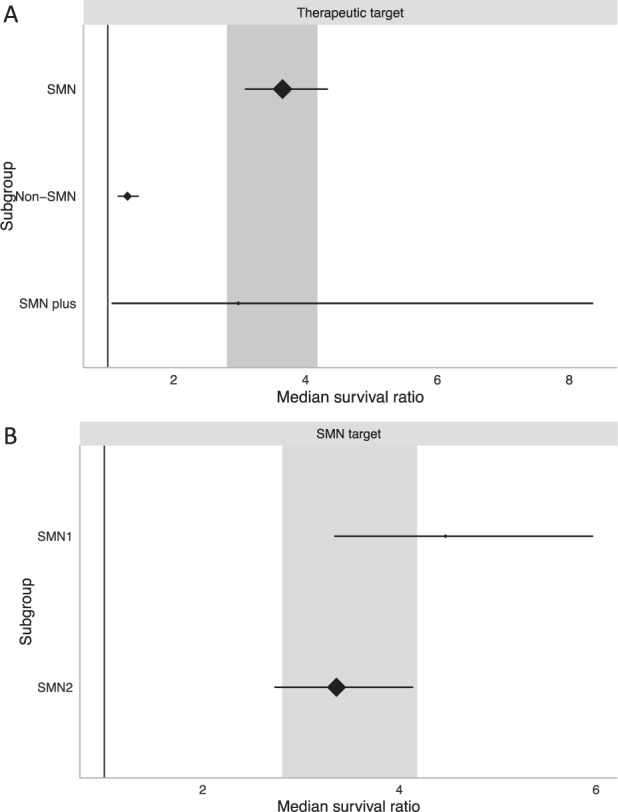
Stratification by therapeutic target. Significant differences in MSR are apparent dependent on (**A**) overall therapeutic target (MSR *P* < 0.0073) and between (**B**) *SMN1*- and *SMN2*-dependent therapies (MSR *P* < 0.0073). Plot represents mean ± 95% confidence intervals with the size of diamonds representing the number of comparisons within each stratum. The vertical line at MSR = 1 represents a neutral treatment effect. Grey rectangle represents global 95% confidence intervals.

Since SMA is a monogenic disease, augmentation of SMN protein production has been the preferred genetic therapy strategy, however SMN-dependent, SMN-independent and SMN-plus strategies have been reported in the literature, with differing improvements in median survival (*χ*^2^ = 363.02, df = 2; *P* < 0.0073). Augmentation of SMN protein, whether this be through replacement of the *SMN1* gene, or manipulation of *SMN2* splicing, provided the largest survival benefit here and was used in 86% of comparisons included (MSR: 3.65, 95% CI 3.08–4.34; *n* = 134; Fig. 4A). A smaller number of comparisons addressed non-SMN targets: Uba1, Plastin3, PTEN, IGF1, CT1, Stathmin, Stasimon, Myostatin and Synaptotagmin13. These led to a more modest increase in survival (MSR: 1.30, 95% CI 1.15–1.47; *n* = 17; Fig. 4A). Furthermore, when combining SMN-dependent and -independent targets into an SMN-plus strategy, the lifespan of animals fell between that of each constituent therapy (MSR: 2.98, 95% CI 1.06–8.36; *n* = 4; Fig. 4A). However, only 72 animals were treated in this manner in three publications.

When directly comparing *SMN*-dependent therapeutic targets (*χ*^2^ = 507.97, df = 1; *P* < 0.0073) it was seen that *SMN1*-targeted therapies produced an MSR of 4.47 (95% CI 3.34–5.97; *n* = 43; Fig. 4B) compared to *SMN2*-dependent MSR of 3.36 (95% CI 2.73–4.14; *n* = 90; Fig. 4B).

#### Mouse model

Although the search employed in this review aimed to retrieve studies from any SMA rodent species, every publication used a mouse model. Most commonly, the SMNΔ7 model was used, followed by the severe Taiwanese model. Other mouse models were also used but in lower frequencies, so have been grouped into one category here. These other models included *Smn2B/−* (*n* = 5), type II/III Burgheron (*n* = 2), hemi-hybrid (*n* = 1), Burghes’ severe (*n* = 8), SMNRT (*n* = 1), moderate type II (*Smn*^*+/−*^ *SMN2 SMN*Δ7, *n* = 1), neuronal *Smn* deletion (*NSE-Cre* + *Smn*^*F7/F7*^, *n* = 1). Improvements in median survival differed between mouse model sub-strata (*χ*^2^ = 471.05, df = 2; *P* < 0.0073).

**Fig. 5:**
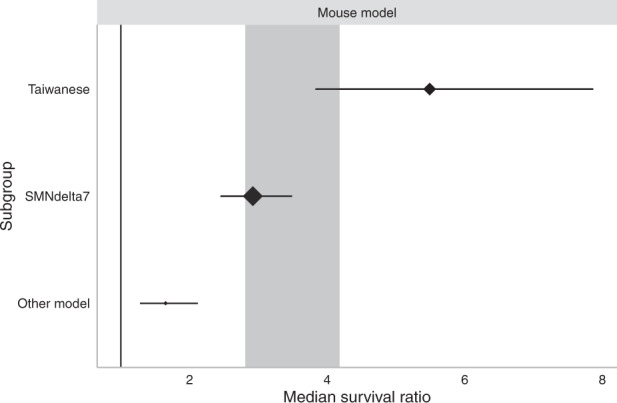
Stratification by SMA mouse model. Significant differences were found between mouse models (MSR *P* < 0.0073). Plots show mean ± 95% confidence intervals with the size of diamonds representing the number of comparisons within each stratum. The vertical line at MSR = 1 represents a neutral treatment effect. Grey rectangle represents global 95% confidence intervals.

Taiwanese mice provide the most severe phenotype within the pure groupings in this review, on average surviving up to 15 days [72]. When genetic therapy was administered to Taiwanese mice a more than five-fold improvement in median survival was found (MSR: 5.49, 95% CI 3.83–7.87; *n* = 41; Fig. 5). SMNΔ7 mice survive ~15–22 days [73] without therapeutic intervention, so are useful when a slightly longer lifespan may reveal more subtle phenotypic benefits of a therapy. SMNΔ7 mice showed a 2.9-fold increase in survival (MSR: 2.92, 95% CI 2.45–3.49; *n* = 96; Fig. 5). Less frequently used mice models showed a more modest increase in survival (MSR: 1.65, 95% CI 1.28–2.12; *n* = 18; Fig. 5).

#### Route of administration

**Fig. 6:**
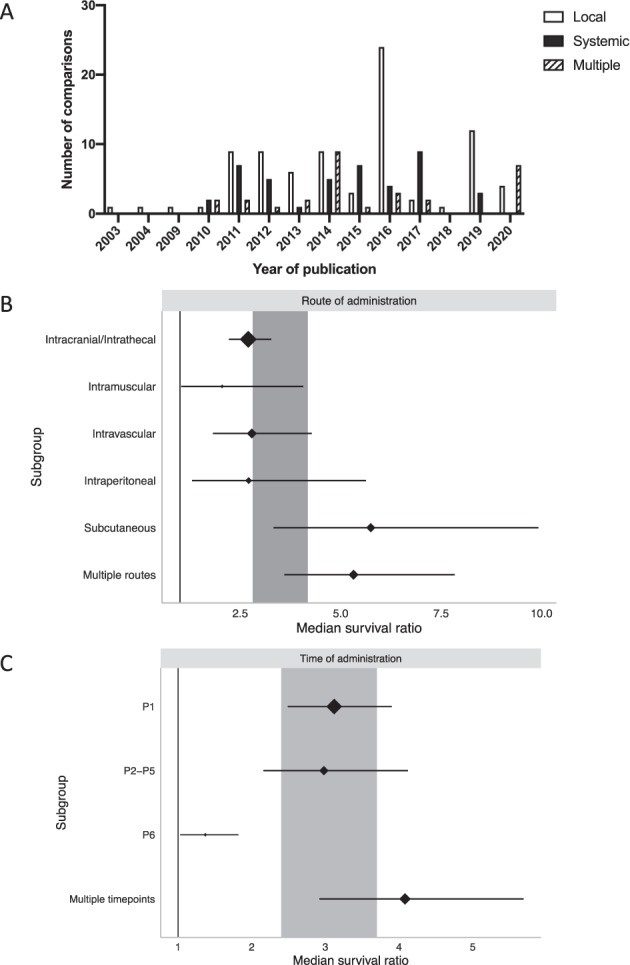
Stratification by route and time of administration of gene therapy. **A** Stratification by year of publication and delivery route shows that local administration remains an often used strategy despite the systemic nature of SMA. **B**, **C** Forest plots demonstrating significant differences in survival data within both route and time of administration strata (MSR *P* < 0.0073). Plots represent mean ± 95% confidence intervals with the size of diamonds representing the number of comparisons within each stratum. The vertical line at MSR = 1 in **B**, **C** represents a neutral treatment effect. Grey rectangles represent global 95% confidence intervals.

Both local (intracranial, intrathecal and intramuscular) and systemic (intravascular, intraperitoneal and subcutaneous) routes of administration were reported in the literature, but significant differences in efficacy were observed between these routes (*χ*^2^ = 422.34, df = 5; *P* < 0.0073). Despite accumulating evidence supporting the systemic nature of SMA, local routes of administration continue being used often (Fig. 6A).

CNS delivery of therapeutics by either intracranial or intrathecal injection was the most commonly used route of administration and was associated with an almost three-fold increase in survival (MSR: 2.70, 95% CI 2.22–3.27; *n* = 77; Fig. 6B). Local, intramuscular delivery more than doubled the lifespan of treated mice (MSR: 2.05, 95% CI 1.03–4.07; *n* = 6; Fig. 6B), highlighting the importance of treating the muscular pathology of SMA. Regarding systemic routes, both intravascular (MSR: 2.79, 95% CI 1.82–4.28; *n* = 22; Fig. 6B) and intraperitoneal (MSR: 2.71, 95% CI 1.30–5.63; *n* = 10; Fig. 6B) delivery were associated with similar survival rates as CNS and intramuscular delivery. Subcutaneous delivery was the third systemic route addressed, providing the largest MSR (5.75, 95% CI 3.33–9.92; *n* = 18; Fig. 6B). Finally, 14% of comparisons investigated used multiple routes of administration within their study. In most cases, these comparisons used intracranial injection in combination with a second route. This led to a 5.32-fold increase in survival (95% CI 3.60–7.84; *n* = 22; Fig. 6B).

#### Time of administration

SMA in its most severe form is a childhood disease with onset in utero. Therefore, early intervention is thought to be key to halting disease progression or providing phenotypic benefit before irreversible pathology occurs [56]. Here, the time of genetic therapy administration greatly impacted the resulting efficacy (*χ*^2^ = 284.93, df = 3; *P* < 0.0073). The need for early intervention is highlighted here with approximately half of comparisons administering genetic therapy on the day of birth. Intervention on P1 leads to a 3.12-fold increased lifespan (95% CI 2.49–3.90; *n* = 83; Fig. 6C). Slightly later intervention within the P2–P5 window provided similar results (MSR: 2.98, 95% CI 2.16–4.12; *n* = 24; Fig. 6C). Administration at P6 or later provided a much lesser, yet still significant benefit (MSR: 1.37, 95% CI 1.03–1.82; *n* = 6; Fig. 6C). Finally, repeated administrations provide the largest increase in survival time seen (MSR: 4.08, 95% CI 2.92–5.69; *n* = 39; Fig. 6C).

#### Post-hoc meta-regression

The above stratified univariate analysis was implemented to identify patterns within data that may suggest aspects of experimental design that lead to the largest survival extensions. However, stratified univariate analyses do not allow for assessment of how variables interact. Therefore at the suggestion of reviewers we added a multivariate meta-regressionin an attempt to identify sources of covariance. We consider this part of our analysis post-hoc and this should be appreciated when interpreting these results.

On proceeding to multivariate meta-regression, there was a significant reduction in the number of studies that could be included due to collinearity (only 69 out of 155 individual comparisons included). In all experimental variables assessed, except time of administration and viral vector dosage, at least one category was dropped from the analysis due to this collinearity (Table 2). When comparing the type of genetic therapy used, none of the oligonucleotide-, viral vector-based or combinatorial approaches could be analysed. Within other variables, Taiwanese mice, intramuscular delivery and SMN-plus therapeutic targets could not be analysed, as well as a complete removal of the *SMN1*- and *SMN2*-specific analysis (Table 2).

When the remaining variables were analysed, the only variable found to be significantly associated with survival outcome was gene target (*P* = 0.0019). SMN-dependent therapies led to an MSR of 5.71 (95% CI 3.54–9.23; *n* = 134), whilst SMN-independent targets had an MSR of 1.28 (95% CI 0.82–2.01; *n* = 17). Otherwise, the model did not suggest a predictive effect of any other variable (Table 2).

In summary, only a small amount of information can be learned from the multivariate meta-regression, other than the fact that there is indeed a large degree of collinearity within this dataset, as is expected in pre-clinical literature.

#### Publication bias

**Fig. 7:**
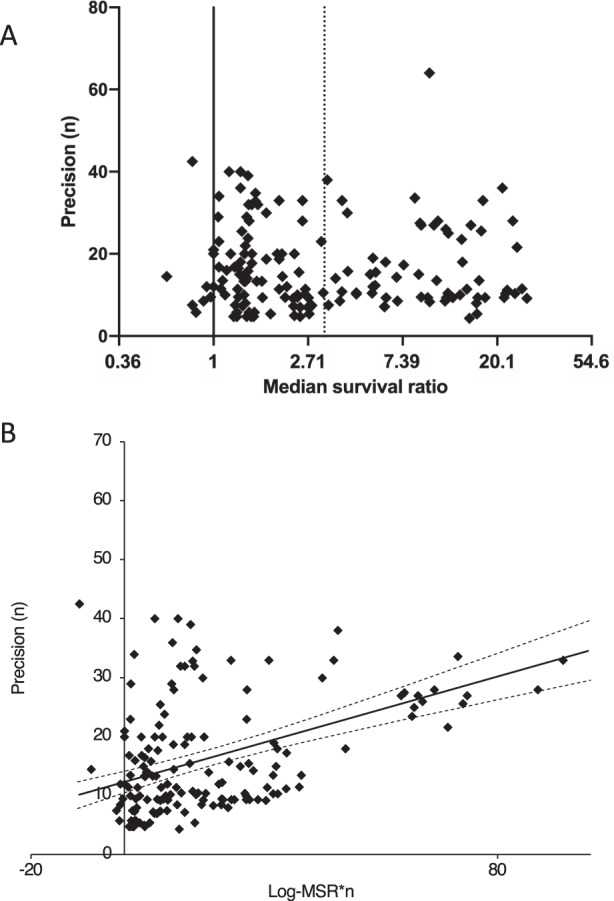
Publication bias in included publications. **A** Funnel plot showing untransformed median survival against study precision (number of animals), with no apparent asymmetry found. **B** Egger’s regression (solid line) revealed positive intercept suggesting imprecise studies showed overstated efficacy. Dotted lines represent 95% confidence intervals.

Publication bias in meta-analyses can occur due to unintentional exclusion of missing data, potentially causing misinformed conclusions to be drawn. Evidence of publication bias can be sought using funnel plots, Egger’s regression and trim and fill analyses (Fig. 7). While there was no obvious asymmetry to the funnel plot, only a small relative number of comparisons reported an effect size <1 (*n* = 7; Fig. 7A). On Egger’s regression we found a positive intercept (Fig. 7B), suggesting the presence of an excess of small, imprecise comparisons overstating efficacy in this analysis. Trim and fill analysis did not suggest the presence of any ‘missing’ publications. However, Trim and fill analysis has been described as a relatively insensitive technique and can be an inadequate method of correcting for publication bias [74].

#### Translation of pre-clinical data to clinical trials

One aim of this systematic review and meta-analysis was to assess how predictive pre-clinical studies can be when translated to clinical trials. Therefore, here we have reviewed *n* = 6 clinical trials; *n* = 5 of which were assessing Spinraza efficacy [75–79] and *n* = 1 assessing Zolgensma [10]. Table 3 details data presented in these studies. Unfortunately, meta-analytic techniques could not be applied to these data for two reasons [1]: no consistent outcomes were reported in all six trials, highlighting the need for consistent outcome reporting across clinical trials, allowing direct comparison of data; and [2] only two out of six studies included control groups [78, 79], meaning statistical methods could not be employed.

**Table 2:** Multivariate meta-regression analysis.

Comparison	Variable(s) included	Variable(s) excluded	Multivariate *P* value
Type of genetic therapy		Oligonucleotide	Not analysed
		Viral vector	
		Combinatorial	
Viral vector dosage	≤e12 vg/kg		0.552
	e13 vg/kg		
	≥e14 vg/kg		
Therapeutic target	SMN-dependent	SMN-plus	0.0019
	SMN-independent		
*SMN*-dependent target		*SMN1*	Not analysed
		*SMN2*	
Mouse model	SMNΔ7	Taiwanese	0.5691
	Other		
Route of administration	Intracranial/Intrathecal	Intramuscular	0.7603
	Intravascular		
	Intraperitoneal		
	Subcutaneous		
	Multiple		
Time of administration	P1		0.1757
	P2–P5		
	≥P6		

Multivariate meta-regression revealed a large degree of collinearity within data, leading to only 69 out of 155 individual comparisons being included in the analysis. Variables that were dropped from the analysis due to this collinearity are shown here. Following exclusion, only therapeutic target was significantly associated with increased survival (*P* = 0.0019; SMN-dependent MSR: 5.71, 95% CI 3.54–9.23; *n* = 134; SMN-independent MSR: 1.28, 95% CI 0.82–2.01; *n* = 17).

**Table 3:** Characteristics of clinical trials using Spinraza and Zolgensma.

Gene therapy	Spinraza					Zolgensma
Study	Chiriboga et al. [74]	Finkel et al. [76]	Finkel et al. [77]	Mercuri et al. [78]	Darras et al. [75]	Mendell et al. [10]
	Phase 1	Phase 2	Phase 3	Phase 3	Phase 1/2	Phase 1
	NCT01780246	NCT01839656	ENDEAR	CHERISH	NCT01703988	NCT02122952
	NCT01494701		NCT02193074	NCT02292537	NCT02052791	
**Participants**	*n* = 28	*n* = 20	Treatment *n* = 80	Treatment *n* = 84	*n* = 28	*n* = 15
			Control *n* = 41	Control *n* = 42		
	Type 2 or 3 SMA	Type 1 SMA	Type 1 SMA	SMA onset after 6 months of age	Type 2 or 3 SMA	Type 1 SMA
**Cohorts**	1, 3, 6 and 9 mg	6 and 12 mg	12 mg	12 mg	3, 6, 9 and 12 mg	6.7 × 10^13^ vg/kg
						2.0 × 10^14^ vg/kg
**Untreated control group**	No	No; compared to natural history cohort *n* = 23	Yes	Yes	No	No; compared to natural history cohort
**Deaths**	0/28	3/20	Treatment = 13/80	0/126	0/28	0/15
			Control = 16/41			
**Serious adverse events**	Treatment = 0/28	Treatment = 16/20	Treatment = 61/80	Treatment = 14/84	Treatment = 5/28	Treatment = 13/15
			Control = 39/41	Control = 12/42		
**HFMSE increase by 3 pts**	1, 3 and 6 mg = 0%	N/A	N/A	Treatment = 57%	Type 2 = 82% and 78%	N/A
	9 mg = 75%			Control = 26%	Type 3 = 19% and 36%	
**HFMSE score**	9 mg = increase of 5.8 points	N/A	N/A	Treatment = increase of 4.9 points	Type 2 = 10.8	N/A
					Type 3 = 1.8 point increase	
**HINE-2 score**	N/A	6 mg = 25%	Treatment = 28%	N/A	N/A	N/A
		12 mg = 100%	Control = 5%			
**CHOP-INTEND score**	N/A	12 mg = 15.2 point increase	Treatment = 71%	N/A	N/A	6.7 × 10^13^ vg/kg = 7.7 point increase
		Natural history = 1.27 point decline	Control = 3% increase ³4 points			2.0 × 10^14^ vg/kg = 24.6 point increase
**Motor milestone response**	N/A	Treatment = 65%	Treatment = 51%	Treatment = 20%	N/A	Treatment = 92%
			Control = 0%	Control = 6%		Natural history = 0%

Outcome measures reported at set follow up time points: Chiriboga et al. [75]: 9-14 months, Finkel et al. [77]: up to 32 months, Finkel et al. [78]: day 394, Mercuri et al. [79]: 15 months, Darras et al. [76]: days 253 and 1050, respectively, Mendell et al. [10]: 24 months. HFMSE, HINE-2, and CHOP-INTEND scores represent change from baseline. Finkel et al. [77] define motor milestone response as “improvement of two or more levels per motor milestone category in at least one category”. Finkel et al. [78] define motor milestone response as “improvement in at least one HINE-2 motor milestone with more categories with improvement than worsening”. Mercuri et al. [79] define motor milestone response as achievement of “≥1 new World Health Organisation motor milestone”.

Overall, 255 affected people were treated and 83 control individuals were included across the 6 studies. 16 deaths were reported in treatment vs 39 deaths in control groups. Serious adverse events were reported in all studies except NCT01780246 and NCT01494701 [75]. In the studies that reported HFMSE (Hammersmith Functional Motor Scale Expanded) scores, some treated patients showed increases by ≥3 points, which are said to be clinically meaningful. HINE-2 (Hammersmith Infant Neurological Examination section 2) and CHOP-INTEND (Children’s Hospital of Philadelphia Infant Test of Neuromuscular Disorders) scores also increased following treatment. Half of the studies reported motor milestones in treated patients compared to control groups, all showing improved responses in treatment groups.

## Discussion

In this meta-analysis of 51 publications, containing data from 2573 animals, we found that overall, genetic therapies led to approximately a three-fold increase in median survival. Stratified meta-analysis suggested a significant impact of type of therapy, mouse models, time and route of administration on perceived treatment effect. To our knowledge, this is the first quantitative meta-analysis of published literature of genetic therapy for SMA. Two other systematic reviews [80, 81] were found, but neither analysed survival benefits. Van der Bent et al. [80] assessed ASO use in heritable neurodegenerative or neuromuscular disorders, including SMA, however, the only data quantitatively analysed pertained to Duchenne Muscular Dystrophy. Qomi et al. [81] systematically describe the development of multiple SMA therapeutic advances at both pre-clinical and clinical level.

With two genetic therapy agents approved for the treatment of SMA patients, a major question concerns the predictive value of pre-clinical studies of oligonucleotide-based approaches that led to Spinraza and viral vector-based approaches that led to Zolgensma. The mechanism and efficacy of Spinraza have been extensively reviewed elsewhere (see refs. [82–85]). A recent, succinct review [86] of pre-clinical AAV9 gene therapy for SMA highlights multiple animal models, including large animals and non-human primates (NHPs).

We observed a significant improvement of median survival with the use of both oligonucleotide- and viral vector-based approaches, with very similar resulting MSRs (3.33 and 3.18, respectively) in the pre-clinical studies analysed in this manuscript. A recent paper [87] compared the Zolgensma NCT02122952 [10] and Spinraza ENDEAR NCT02193074 [78] clinical trials and found that patients treated with Zolgensma had a 20% higher probability of prevented death, than patients treated with Spinraza (risk ratio 1.2, 95% CI 1.1–1.3). At the last follow up visit in each trial, 100% of Zolgensma patients were alive, whereas only 84% of Spinraza patients were [87]. However, several limitations of this comparative study should be noted [88]. Trial design (including aspects such as multi- versus single-centre design) and baseline characteristics of treated patients (including age at first dose, mean disease duration and mean motor function score) were not adjusted for in the number needed to treat analysis conducted in this study and therefore potentially confound any conclusions drawn. Baseline characteristics show a more severe, older patient population in the ENDEAR trial, perhaps explaining the apparent lower efficacy concluded by Dabbous et al. [87]. It should also be stated that the authors of this comparative study were Avexis employees.

With regards to an oligonucleotide plus viral vector combinatorial approach, the efficacy of combinatorial treatment here in fact led to the most pronounced survival benefit, but data are minimal as only two publications [70, 71] attempted this. Within the clinical setting, three patients from the Zolgensma NCT02122952 [10] clinical trial are now said to be also being treated with Spinraza, but data from these patients are not available at the time of writing. The phase 4 RESPOND clinical trial has been designed to administer Spinraza to infants previously treated by Zolgensma, who may have responded sub-optimally to the viral vector therapy. Enrollment is due to begin globally in 2021.

Mendell et al. [10] presented data from two cohorts of Zolgensma treatment in their clinical trial; one low dose of 6.7 × 10^13^ vg/kg and one high dose of 2.0 × 10^14^ vg/kg. Their rationale for using these two dosages was that in selected pre-clinical models, the low dose doubled survival, but the high dose led to a 250 day survival compared to 15 day control survival [47–49, 89]. In contrast, in the preclinical data (which entailed a broader selection of paradigms), a lower dose (e13 vg/kg; *n* = 30) was in fact associated with greater efficacy than higher (≥e14 vg/kg; *n* = 34) viral vector dosage. However, there were some differences in experimental design so this finding may be influenced by unaccounted confounders. Respectively, e13 and ≥e14 vg/kg dosage strata showed differences in use of mouse model (83% vs 73.5%: SMNΔ7 mice), route of delivery (43% vs 58%: ICV) and transgene (10% vs 5.9%: codon-optimised *SMN1*). Raw median survival ranges of treated SMA animals also differ between the two strata (e13 vg/kg: 7.9–346 days; ≥e14 vg/kg: 9–250 days) in favour of the e13 vg/kg dosage. Biologically, it may be possible that transgene saturation had occurred in the higher dose. Potentially, if SMN protein was already produced at supraphysiological levels at e13 vg/kg, as suggested within Passini et al. [46, 55], Benkhelifa-Ziyyat et al. [53], and Dominguez et al. [49], increasing viral vector dosage beyond this in rodent models may not lead to a further increase in survival and perhaps be even less efficacious. It has also recently been shown that supraphysiological levels of SMN leads to a late-onset gain of toxic function phenotype caused by disrupted snRNP biogenesis and neuroinflammatory-linked transcriptome changes [90].

It is also important to note the potential safety concerns over high-dose AAV vector therapies. A recent report has highlighted that 34% of Zolgensma-treated patients across five clinical trials, a managed access programme and commercial use suffered some degree of hepatotoxicity [91]. Subacute liver failure has been reported in at least two cases of high-dose Zolgensma-treated patients (6.25 × 10^14^ and 11.55 × 10^14^ total vector genomes) [92]. Not limited to the treatment of SMA, two children enrolled in the ASPIRO clinical trial for X-linked myotubular myopathy have recently died of sepsis following AAV8_AT-132 therapy [93]. Both boys were in the high-dose cohort administering 3 × 10^14^ vg/kg, which equates to a dose in excess of 4 × 10^15^ total vector genomes [94]. Bearing these concerns in mind, it is crucial to design strategies that mitigate these risks and investigate aspects of vector design that could negate the need for such high viral loads.

Pre-clinical assessment of therapeutic efficacy can be heavily influenced by the disease model in which the therapy is applied. For SMA, many mouse models exist with varying phenotypes ranging from severe to more mild phenotypes mimicking type II or III SMA. Although SMA mouse models are the most commonly used, models from other species are also available. Increasingly, more non-mouse studies are appearing in the literature using zebrafish [58, 95, 96], cats [97], pigs [98] and NHPs [48, 99]. However, these were not included in this review in order to appreciate the effects of study design and quality more reliably.

Here, we observed greater survival benefits when genetic therapy was given to Taiwanese mice than in treated SMNΔ7 mice. Furthermore, bimodal survival curves were reported in at least three comparisons assessing AAV-treated SMNΔ7 mice, perhaps suggesting there is a population of animals whose phenotype cannot be ameliorated by AAV-mediated therapeutics. In these publications, the first group of animals died before ~1 month of age (17–27 days [46], 25–35 days [51] and 27–32 days [49]). The four seminal papers [46–49] first describing AAV_*SMN*-mediated increase in survival all used SMNΔ7 mice. These papers cited the choice of SMNΔ7 mice due to the robust phenotype including an approximate 2-week lifespan, loss of MNss, skeletal muscle atrophy and progressive body weight decline. The SMNΔ7 model had also widely been used in previous pharmacological efficacy studies due to this phenotype [47].

It is possible that the mix of different SMA severities within the collated group of less frequently used mouse models contributed to the lower survival benefit seen. For example, Burghes’ severe mice survive 4–6 days on average, whilst type II/III models may survive into adulthood. We did not attempt to delineate a severe and a mild group from these other models to avoid inflicting bias when categorising less frequently used models.

The manner in which a therapy is delivered is important to both patients and clinicians. Spinraza is delivered through an intrathecal injection, whilst Zolgensma is intravenously administered. Lumbar puncture in young children, such as those under the age of 6 months with type I SMA, especially those with severely distorted spines, can be distressing and has associated risks not seen with other modes of delivery. Mercuri et al. [79] observed 9% of adverse events were associated with lumbar puncture 24 h post Spinraza delivery, rising to 15% at 168 h and these were at least 5% higher than in the sham lumbar puncture control group. Intrathecal drug delivery, in bypassing the blood–brain barrier, provides good CNS penetrance. SMN protein levels augmented by Spinraza are restored in anterior horn cells, but all tissues outside of the CNS are unaffected. Similarly, restricting SMN production to neurons through transcriptional targeting with the synapsin promoter in AAV9 led to reduced rescue in the SMNΔ7 mouse model ([68]; this study was excluded from our meta-analysis due to being outside the cut-off date). CNS-targeted therapies may improve survival of SMA patients by preventing MN degeneration and its consequences, but hitherto masked peripheral organ damage may become increasingly prevalent in the clinical phenotype, presenting yet unknown burdens. Because of this issue, systemic gene delivery has been a point of interest within recent SMA research.

The definition of a therapeutic window in which administration of a therapeutic agent provides clinical benefit is important, particularly in a disease like severe SMA whose genesis is in utero. Studies have aimed to define this window [56]. For some time, it has been thought that the pre-clinical therapeutic window for SMA exists from the day of birth to ~3 days afterwards. Recently an AAV9_*SMN* therapy for SMA has been delivered to mice in utero for the first time, with results indicating a significant increase in survival compared to untreated animals [64], highlighting the potential of fetal genetic therapy for SMA too. Here, similar efficacies can be seen when genetic therapy was administered on the day of birth, or between P2 and P5 (MSR: 3.12 and 2.98, respectively). Pre-symptomatic delivery of treatment may prevent development of the SMA phenotype and the irreversible damage that accompanies this, perhaps due to the deficiency being corrected during the period of neuromuscular junction maturation [4]. Later delivery, on or after P6, causes a dramatic decrease in efficacy (MSR: 1.37), consistent with current knowledge that symptom onset begins at approximately this time, such as reduction in body weight from P6 onwards in SMNΔ7 mice [47]. Administering repeated doses of genetic therapy increased MSR further compared to the leading single time point (P1). Of the comparisons that administered genetic therapy at multiple time points, 55% of these used an oligonucleotide approach. This is consistent with the delivery of Spinraza in the clinic, where intrathecal injections are given every four months during the stable dosing phase, in contrast to a single dose of Zolgensma.

Monogenic diseases such as SMA are prime candidates for gene replacement therapies, thus it is not surprising that 86% of comparisons reviewed here used an *SMN*-dependent approach and these were associated with the greatest survival improvements. It is also reasonable that replacement of the missing *SMN1* gene would provide more benefit than augmentation of SMN protein produced by targeting *SMN2*, as we have identified via a 1.3-fold difference between MSRs (4.47 and 3.36, respectively). Nevertheless, the contributions of disease modifiers are increasingly being linked with the alteration of SMA phenotypes. *Plastin3* and *NCALD* are protective modifiers of SMA in humans, although further modifiers have been found in animals [100]. When studying the interactome of SMN and SMA disease modifiers, non-SMN proteins have been discovered as potential therapeutic targets. Non-SMN targets have been reviewed excellently elsewhere [3, 101]. Within this meta-analysis 17 comparisons targeted non-SMN proteins with a 44% increase in survival, albeit lower than SMN-dependent survival.

Further evidence for the use of non-SMN targets to treat SMA is available from non-genetic therapy clinical trials formerly evaluating Olesoxime (now discontinued) and currently assessing Reldesemtiv and SRK-015. These drugs aim to combat oxidative stress in mitochondria, muscle fatigue and improve muscle strength, respectively. With regards to addressing both SMN and non-SMN targets, also known as a SMN plus strategy, it is possible to use ASOs alone [23], viral vectors alone or both [70], to express or modify each target. Here, these approaches led to an MSR of 2.98, higher than that of non-SMN-dependent strategies. Many further publications were found during the literature search using SMN plus strategies, but were ineligible to be included as they modified the non-SMN target via germline transgenesis, instead of gene therapy delivered in vivo. Two example studies showed promising results with transgenic animals (*Smn*^−/−^ *SMN2*^tg/0^ *Chp1*^vac/wt^) plus *SMN2* targeting ASOs [100] as well as transgenic animals (*Smn*^−/−^ *SMN2 KLF15* Mtg) plus viral vectors [102]. A further publication reported administration of scAAV9_*DOK7*, a neuromuscular junction protein, to *Smn2B/−* mice, leading to a significant increase in median survival by 1 day, however, this was not included in the meta-analysis due to being identified after the pre-specified search cut-off date [103].

Limitations of meta-analytic statistics are, of course, present. Risk of bias was prevalent in a random sample of publications describing in vivo research [104]; coupled with a proclivity for the “file drawer problem”, selective publication of positive results, published treatment efficacies are generally inflated. As conventional meta-analytic techniques could not be used with median survival data, an estimate of standard error was made using sample sizes, weights and inter-study variance so that a random effects model could be implemented [12, 13]. While not as precise as the gold-standard hazard ratio model used in clinical meta-analyses, we believe this model approach to be valid in the context of the limitations in the data. We have tested the same dataset multiple times and have managed the risk of type 1 errors by using Bonferroni correction.

A significant limitation in this meta-analysis is the application of a univariate model, which does not allow for assessment of how variables interact. Given the varied study designs seen in small animal literature, covariance is generally an issue in preclinical meta-analyses. An example from the data presented here highlights this: all but three comparisons administering genetic therapy via subcutaneous delivery used Taiwanese mice as the chosen model. Both of these two sub-strata showed very high MSRs. With a univariate approach it is impossible to determine which of these factors is influential. On this basis, we strongly suggest that these results should be interpreted with caution and considered hypothesis-generating only: resulting questions should be investigated through the conduction of high-quality prospective studies.

Multivariate meta-analysis techniques have been described in preclinical literature [105, 106], but their adoption with median survival data has not yet been fully validated. Here, we implemented these techniques in an attempt to identify the variables that most influence survival outcomes following SMA genetic therapy. It is important to note that this was implemented as a post-hoc addition following reviewer comments and was not included in the analysis protocol. However, the results from this analysis were largely inconclusive. Over half of the comparisons included in the original stratified univariate analysis were excluded from the multivariate meta-regression due to a high degree of collinearity, meaning that comparison of most variables did all include all possible options presented in the primary literature. The only variable found to be associated with a significant impact on survival outcome was therapeutic target, but again, only SMN-dependent and non-SMN therapies could be analysed as SMN-plus approaches were excluded; significant exclusions (such as the lack of the Taiwanese model and intramuscular delivery) and low sensitivity of multivariate meta-regression should be borne in mind. Multivariate meta-regressions are known to have a low power to detect associations [107] and since their use with median survival data has not been well studied, we suggest the results of this analysis be interpreted with caution.

With the availability of Spinraza and Zolgensma (and Evrysdi), SMA is the most successfully treated genetic neuromuscular disease. Multiple factors are likely responsible for this: an extensive population of people affected, considerable knowledge of the natural history, a thorough understanding of the genetic basis which has provided various therapeutic strategies, a small cDNA that can be easily packaged, suitable routes of local or systemic delivery, a variety of cellular and animal models for testing, an understanding of the therapeutic window, and the availability of clinical scales or phenotypes that can be measured, among others. The possibility of combinatorial therapy and the existence of a significant pipeline of treatments undergoing pre-clinical and clinical development support further optimism. Not all these factors are relevant to other genetic diseases, but SMA has been a trailblazer and has facilitated the application of similar technology to other CNS diseases. It seems also clear that while Spinraza and Zolgensma improve the SMA phenotype, they are not cures. Further research is therefore necessary to improve therapeutic outcomes in SMA.

## Conclusions

Genetic therapies have demonstrated therapeutic efficacy for SMA in the clinic. This systematic review and meta-analysis of pre-clinical research has confirmed that genetic therapies can significantly prolong survival, but also that experimental design has a fundamental influence on perceived study outcome. Furthermore, pre-clinical results appear to correlate well with clinical experience of Spinraza and Zolgensma. However, pre-clinical data are typically at high risk of bias and single paradigms have not reliably predicted translational efficacy. Our conclusions should be borne in mind when conducting further pre-clinical studies of other candidate SMA treatments, as well as more generally small animal research of genetic therapies.
