# Correction: Systematic review and meta-analysis determining the benefits of in vivo genetic therapy in spinal muscular atrophy rodent models

**DOI:** 10.1038/s41434-022-00377-8

**Published:** 2022-11-28

**Authors:** Ellie M. Chilcott, Evalyne W. Muiruri, Theodore C. Hirst, Rafael J. Yáñez-Muñoz

**Affiliations:** 1 AGCTlab.org, Centre of Gene and Cell Therapy, Centre for Biomedical Sciences, Department of Biological Sciences, School of Life Sciences and Environment, Royal Holloway University of London, TW20 0EX London, UK; 2 Department of Neurosurgery, Royal Victoria Hospital, Belfast, BT12 6BA UK; 3 Present Address: Institute for Women’s Health, UCL, 86-96 Chenies Mews, London, WC1E 6HX UK

**Keywords:** Genetic transduction, Gene expression

Correction to: *Gene Therapy* (2021) 29:498-512 10.1038/s41434-021-00292-4, published online 06 October 2021

In this article, the title and caption of Table 3 were incorrectly given. They should have been as below.

Table 3. Characteristics of clinical trials using Spinraza and Zolgensma.

Outcome measures reported at set follow-up time points: Chiriboga et al. [75]: 9-14 months, Finkel et al. [77]: up to 32 months, Finkel et al. [78]: day 394, Mercuri et al. [79]: 15 months, Darras et al. [76]: days 253 and 1050, respectively, Mendell et al. [10]: 24 months. HFMSE, HINE-2, and CHOP-INTEND scores represent change from baseline. Finkel et al. [77] define motor milestone response as “improvement of two or more levels per motor milestone category in at least one category”. Finkel et al. [78] define motor milestone response as “improvement in at least one HINE-2 motor milestone with more categories with improvement than worsening”. Mercuri et al. [79] define motor milestone response as achievement of “≥1 new World Health Organisation motor milestone”.

